# Dartmouth College Honors—Dr. R. R. Andrews

**Published:** 1892-08

**Authors:** 


### DARTMOUTH COLLEGE HONORS—DR. R. R. ANDREWS.

The honorary degree of Master of Arts (A.M.) was conferred on Dr. Andrews by Dartmouth College at the commencement, June 3, in recognition of his "work in microscopy and dental histology."

The dental profession so thoroughly appreciates the results of Dr. Andrews's labor, that the feeling will be universal that it is an honor nobly earned and justly given.

Universities and colleges, where they refuse to grant honorary professional degrees, still adhere to the old custom of conferring the degrees of Master of Arts, Doctor of Divinity, and Doctor of Laws upon "strangers of distinguished reputation," and in recognition of advanced work. This will, probably, be continued for an indefinite period.

